# Heterologous Prime-Boost Vaccination Enhances *Ts*Pmy’s Protective Immunity against *Trichinella spiralis* Infection in a Murine Model

**DOI:** 10.3389/fmicb.2017.01394

**Published:** 2017-07-21

**Authors:** Lei Wang, Ximeng Sun, Jingjing Huang, Bin Zhan, Xinping Zhu

**Affiliations:** ^1^Beijing Tropical Medicine Research Institute, Beijing Friendship Hospital, Capital Medical University Beijing, China; ^2^Department of Medical Microbiology and Parasitology, School of Basic Medical Sciences, Capital Medical University Beijing, China; ^3^Department of Pediatrics, National School of Tropical Medicine, Baylor College of Medicine, Houston TX, United States; ^4^Research Centre of Microbiome, Capital Medical University Beijing, China

**Keywords:** *Trichinella spiralis*, vaccine, prime-boost, mucosal immunity, *Salmonella typhimurium*

## Abstract

*Ts*Pmy is a paramyosin expressed by parasitic *Trichinella spiralis* and confers a protective immunity when its recombinant protein or DNA was used as an immunogen. To improve its immunogenicity and vaccine efficacy, we conducted a heterologous prime-boost strategy by orally delivering one dose of *Ts*Pmy DNA carried by attenuated *Salmonella typhimurium* (SL7207), followed by two doses of recombinant *Ts*Pmy intramuscularly. This strategy effectively induced intestinal mucosal sIgA response and an enhanced and balanced Th1/Th2 immune responses that improve protection against *T. spiralis* larval challenge, with 55.4% muscle larvae reduction and 41.8% adult worm reduction compared to PBS control. The muscle larvae reduction induced by heterologous prime-boost regimen was significant higher than that induced by the homologous DNA or protein prime-boost regimens, which could act as a practical prophylactic approach to prevent *T. spiralis* infection.

## Introduction

Trichinellosis remains endemic in 66 countries with estimated 11 million people infected with *Trichinella spiralis* around the world (Dupouy-Camet, 2000). Only in China, total 32 outbreaks of human trichinellosis with 2,215 cases and 15 deaths have been reported during 2000 to 2009 (Wang et al., 2006; Cui et al., 2011). *T. spiralis* infects not only humans but also a wide range of domestic and wild animals that becomes major source of human infection through eating undercooked meat contaminated with infected larvae. The infected domestic animals such as porcine as major source for human infection make it difficult to control endemic of trichinellosis. There is a need to develop vaccine as an alternative approach to control the infection.

Many efforts have been made to develop vaccines against trichinellosis during the past decades, including vaccines based on worm crude larval extracts, recombinant proteins, DNA or multiple-epitope peptide which induced certain extent of protective immunity in animal models (Gu et al., 2008; Wang et al., 2009; Wei et al., 2011). However, these traditional vaccines and immunization regimen present challenges with insufficient protective effect against *T. spiralis* infection. Except for selecting the proper vaccine candidate which induces the best protective immunity, it is also very important to optimize the immunization regimen such as immunization schedule, route of administration, vaccine dose and delivery systems as well as adjuvants in order to achieve the best protective efficacy (Kardani et al., 2016).

*Trichinella spiralis* is an intestinal nematode that develops to adult worm in small intestine in which male and female worms mate and female worms lay newborn larvae. The later penetrate the intestine and migrate to muscle tissue where they form cysts. Thus, intestinal mucosa is the first crucial barrier against *Trichinella* infection and larva migration. The mucosal immune response in the gut is likely to be important in protecting the host against *T. spiralis* infection (Picherot et al., 2007). In our previous study, attenuated *Salmonella typhimurium* (SL7207) was used as delivery system to orally deliver a DNA vaccine of *Ts*Pmy (Wang et al., 2016), a leading vaccine candidate for trichinellosis (Yang et al., 2008, 2010; Wei et al., 2011; Gu et al., 2013). Mice orally inoculated with *Ts*Pmy/*Salmonella* DNA vaccine displayed strong intestinal mucosal and systemic immune responses which resulted in 44.8% reduction in adult worm and 46.6% reduction in muscle larvae against *T. spiralis* larval challenge (Wang et al., 2016).

Heterologous prime-boost immunization regimen, primed by DNA vaccine and boosted by recombinant protein, has be proven to induce stronger cellular and humoral immunity (Jackson et al., 2014; Kardani et al., 2016) and the better protection against some infectious diseases such as *Leishmania infantum* (Shahbazi et al., 2015), *Toxoplasma gondii* (Han et al., 2017), *Plasmodium yoelii* (Cabrera-Mora et al., 2016), and *Schistosoma japonicum* (Dai et al., 2017). In present study we used *Salmonella*-delivered *Ts*Pmy DNA as prime immunization followed by two boost immunizations with expressed recombinant *Ts*Pmy protein (r*Ts*Pmy) in order to enhance *Ts*Pmy’s immunogenicity and protective efficacy against *T. spiralis* infection in a murine model.

## Materials and Methods

### Ethics Statement

Female BALB/c mice, 6–8 week-old and free of specific pathogens, were obtained from Laboratory Animal Services Center of Capital Medical University (Beijing, China). All mice were maintained under specific pathogen-free conditions at an appropriate temperature and humidity. Experimental procedures were consistent in strict with the NIH Guide for the Care and Use of Laboratory Animals, that were carried out in accordance with the protocols approved by the Capital Medical University Animal Care and Use Committee (Permission No: 2012-X-108).

### *Ts*Pmy DNA Vaccine

The DNA encoding full-length *Ts*Pmy (GenBank ID: EF429310) was cloned into the eukaryotic expression vector pVAX1, and the recombinant pVAX1-*Ts*Pmy plasmid DNA was transformed into an attenuated *S. typhimurium* (SL7207) which was aroA mutant strain with 2337–2365 derivative *his*G46 DEL407 (aroA::Tn*10* {Tc-s}) by electroporation (2.5 kV, 25 μF, and 200 Ω) as an oral DNA vaccine (SL7207/pVAX1-*Ts*Pmy), the transformants were selected on plates with 50 μg/ml kanamycin and identified by PCR amplification and restriction enzyme digestion. The PCR products were then confirmed by DNA sequencing as previous described (Wang et al., 2016).

### Recombinant *Ts*Pmy Protein (r*Ts*Pmy)

DNA encoding *Ts*Pmy without signal peptide was cloned into the expression vector pET-28a (+). The recombinant *Ts*Pmy protein (r*Ts*Pmy) was expressed and identified in an *Escherichia coli* (BL21) expression system in our laboratory. The r*Ts*Pmy with a His-tag at the C terminus was expressed in *E. coli* under 1 mM IPTG induction and purified with Ni-affinity chromatography (Qiagen, United States) as previously described (Yang et al., 2008).

### Parasites

*Trichinella spiralis* (ISS 533) parasites used in this study were originally isolated from an infected pig in Heilongjiang, China and maintained by serial passage in female ICR mice in our laboratory (Cui and Wang, 2001). The adult worms from the small intestines of infected mice were isolated 5 days post-infection (dpi). *T. spiralis* muscle larvae (ML) from infected mice were recovered at 42 dpi via a previously described pepsin-hydrochloric acid digestion method (Gamble et al., 2000).

### Immunization Regimens and Sample Collection

BALB/c mice were randomly divided into six groups and each group was composed of 40 animals. For the heterologous prime-boost group (DNA+Protein), each mouse was inoculated orally with 1 × 10^8^ attenuated *S. typhimurium* SL7207 containing pVAX1-*Ts*Pmy (SL7207/pVAX1-*Ts*Pmy) as previously described (Wang et al., 2016) and then boosted intramuscularly twice with 25 μg r*Ts*Pmy emulsified with an equal volume of the water-in-oil adjuvant ISA 50V2 (SEPPIC, France) at 2-weeks interval. For DNA or recombinant protein vaccination groups, mice were immunized either orally with 1 × 10^8^ bacteria of SL7207/pVAX1-*Ts*Pmy (DNA) or intramuscularly with 25 μg of r*Ts*Pmy formulated with ISA 50V2 (Protein) for three times at 2-weeks interval. The other three control groups were given with either the same number bacteria with empty vector of SL7207/pVAX1 orally (Vector), or PBS alone (emulsified with ISA 50V) intramuscularly for three times, or first oral vector followed with two PBS intramuscularly to mimic heterologous prime-boost regimen (Vector+PBS) as shown in **Table 1**.

**Table 1:** Immunization groups (immunization route).

Groups	Prime	1st boost	2nd boost
DNA+Protein	SL7207/pVAX1-*Ts*Pmy(orally)	r*Ts*Pmy(intramuscularly)	r*Ts*Pmy(intramuscularly)
DNA	SL7207/pVAX1-*Ts*Pmy(orally)	SL7207/pVAX1-*Ts*Pmy(orally)	SL7207/pVAX1-*Ts*Pmy(orally)
Protein	r*Ts*Pmy(intramuscularly)	r*Ts*Pmy(intramuscularly)	r*Ts*Pmy(intramuscularly)
Vector+PBS	SL7207/pVAX1(orally)	PBS(intramuscularly)	PBS(intramuscularly)
Vector	SL7207/pVAX1(orally)	SL7207/pVAX1(orally)	SL7207/pVAX1(orally)
PBS	PBS(intramuscularly)	PBS(intramuscularly)	PBS(intramuscularly)

Before immunization and 1 week after each immunization, five mice from each group were sacrificed; the lavage fluid of intestines, serum, mesenteric lymph nodes (MLNs), and spleen were collected from each mouse to examine the levels of mucosal, humoral and cellular immune responses, as previously described (Wang et al., 2016). All animals were daily monitored for general appearance, abnormal locomotion, eye abnormality, labored breathing, head tilt, hyperactivity, lethargy, paresis, lameness, paralysis, ruffled fur, and tremors. Mice would be euthanized immediately by CO_2_ inhalation if there was any severe sign of abnormality including bleeding diarrhea, leg injuries or in moribund condition.

### Humoral Antibody Responses in Systemic and Mucosal Immunity

The levels of *Ts*Pmy-specific total IgG, IgG1, and IgG2a antibodies in the sera of the immunized mice were detected via a modified ELISA using r*Ts*Pmy coated on plates as described previously (Yang et al., 2010). Briefly, microtiter plates were coated with r*Ts*Pmy (1 μg/ml) at 4°C overnight. Then, 100 μl of sera samples diluted in PBS at 1:1000 was added to each well. HRP-conjugated goat-anti-mouse IgG, IgG1, or IgG2a antibodies were added as secondary antibodies. 3,3′,5,5′-tetramethylbenzidine was used as a substrate and the reactions were stopped with 50 μl per-well of 2 M H_2_SO_4_. Absorbance at 450 nm was measured with a microplate reader. All samples were run in triplicate.

To evaluate the secreted IgA (sIgA) level, the 10 cm interior small intestine from each sacrificed mouse was washed twice with 2 ml of cold PBS. After centrifugation at 800 × *g* for 10 min, the supernatants were harvested. Intestinal total sIgA was quantified with sandwich-type ELISA using rat anti-mouse IgA antibody as the capture antibody. The specific *Ts*Pmy-specific sIgA in intestinal lavage fluid of each mouse was measured by standard ELISA using r*Ts*Pmy as antigen coated on plates, respectively, as described previously (Yang et al., 2010; Wang et al., 2016).

### Cellular Cytokine Assay

To examine the specific cellular immune responses against r*Ts*Pmy, the cytokine profiles in splenocytes and MLN cells from immunized mice were analyzed upon re-stimulation with the r*Ts*Pmy antigen *in vitro* using a modified ELISPOT assay (BD Bioscience, United States). In brief, spleen and the MLN cells were isolated aseptically from mice 7 days after each immunization and the single lymphocyte suspensions were prepared in RPMI-1640. 1 × 10^6^ lymphocytes were added to each well of plates pre-coated with the capture antibody (anti-mouse IFN-γ, IL-2, IL-4, IL-6, and IL-10) at 1: 200 dilutions in PBS and incubated at 4°C overnight. After stimulation with 1 μg/ml of r*Ts*Pmy for 48 h, the biotinylated secondary antibodies were added for 2 h, then incubated with streptavidin-HRP for 1 h and developed with 100 μL of 3-amino-9-ethylcarbazole substrate solution (BD Biosciences, United States) for 30 s–5 min. The spots secreted with IFN-γ, IL-2, IL-4, IL-6, and IL-10 were counted as spot forming units (SFUs) with a CTL ELISPOT reader and analyzed using ImmunoSpot image analyzer software v4.0.

### Challenge Experiment

Two weeks after the final boost, the remaining 20 mice in each group were orally challenged with 500 *T. spiralis* ML per mouse. Ten mice from each group were euthanized 5 days after challenge and the adult worms were collected and counted. The left 10 mice were euthanized 42 days after challenge infection and the muscle larva were collected and counted. The adult worm and muscle larvae reduction in immunized mouse groups was calculated compared with those from the PBS control mice.

### Statistical Analysis

One-way ANOVA was used for data comparison among different groups with SPSS version 17.0 software and expressed as the means ± standard deviation (SD), *p* < 0.05 was regarded as statistically significant.

## Results

### Systemic Humoral Response Elicited by Different Regimen

**FIGURE 1:**
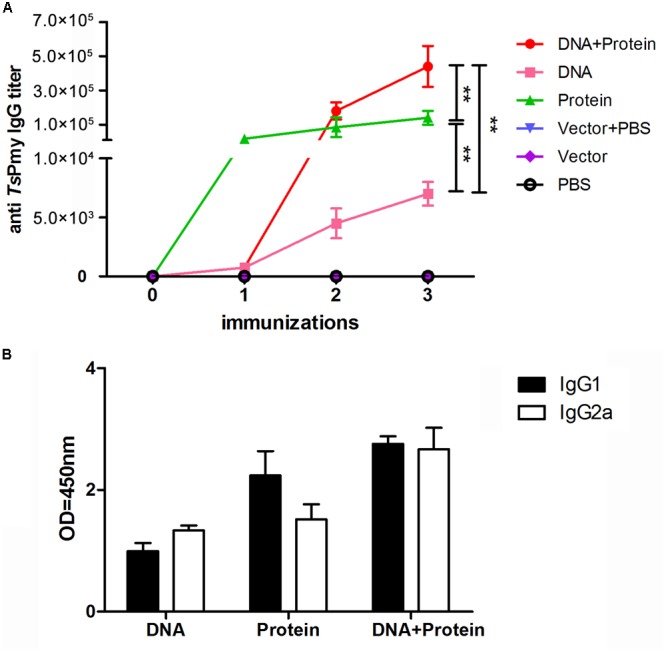
Analysis of sera antibody responses in mice immunized with *Ts*Pmy in different regimens. **(A)** Specific anti-*Ts*Pmy IgG levels in the sera of immunized mice at different immunization time points for five individual mice. **(B)** The levels of the subclass IgG1 and IgG2a when sera were diluted at 1:200. The OD values shown for each group are the mean ± standard deviation (SD) of the antibody levels. ^∗∗^*p* < 0.01 between two groups.

Mouse sera collected 1 week after each immunization were used to determine the antibody levels of anti-*Ts*Pmy IgG and its subtype (IgG1 and IgG2a). The anti-*Ts*Pmy specific IgG was detected in sera of mice immunized with DNA+Protein, DNA, or Protein alone after first immunization compare to the three empty control groups. The anti-*Ts*Pmy IgG reached the highest titers after three immunizations. Specifically, mice immunized with protein prime-boost (Protein) produced significant high titers of anti-*Ts*Pmy IgG after the first immunization and remained the plateau after two boosts. The anti-*Ts*Pmy IgG level in mice of DNA+Protein prime-boost group was significantly increased after first boost with r*Ts*Pmy and reached to even higher titer than mice in Protein prime-boost group after three immunizations. The mice immunized with DNA prime-boost produced lower anti-*Ts*Pmy IgG titer than other two vaccine groups after three immunizations. However, none of the mice in control groups received Vector+PBS, Vector or PBS alone showed any detectable *Ts*Pmy specific IgG response (**Figure 1A**). Similarly, after three immunizations, mice immunized with DNA+Protein produced highest anti-*Ts*Pmy IgG1 and IgG2a compared to DNA or Protein homologous prime-boost groups and the IgG1 and IgG2a level was proximately equal, indicating balanced Th1/Th2 immune responses were induced in heterologous prime-boost immunization. Mice immunized with Protein prime-boost produced higher IgG1 than IgG2a, indicating predominant Th2 responses. Mice immunized with DNA alone produced lowest anti-*Ts*Pmy IgG1 and IgG2a compared to other two vaccine groups, with IgG2a predominant (**Figure 1B**).

### Intestinal Mucosal sIgA Response

**FIGURE 2:**
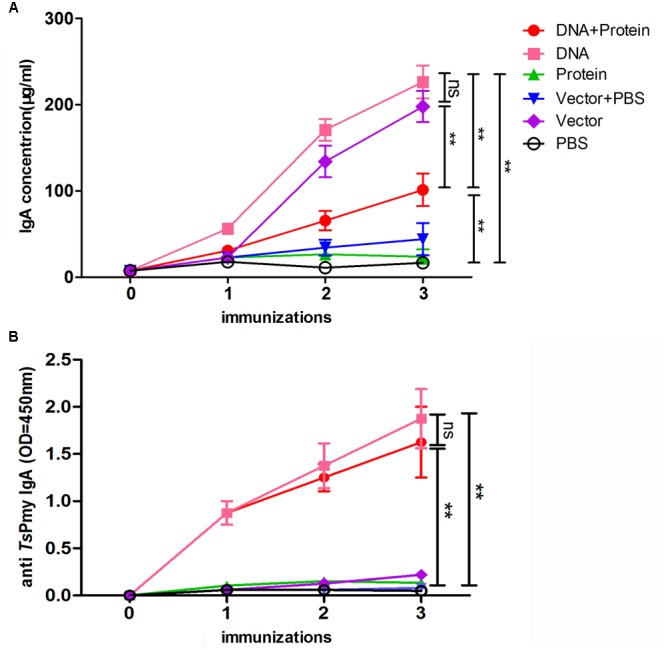
The secretion of total sIgA **(A)** and *Ts*Pmy specific sIgA **(B)** in the lavage fluid of intestines of mice in different regimens measured by modified ELISA. Results are the mean ± standard deviation (SD) for five individual mice per group. The *Ts*Pmy specific sIgA level is shown using OD_450_ value. ^∗∗^*p* < 0.01 between two groups; ns, not significant.

The intestinal total sIgA were measured by a sandwich ELISA and anti-*Ts*Pmy specific sIgA were evaluated using an r*Ts*Pmy coated plate in intestinal mucosal washings. Intestinal total sIgA level was significantly increased in the intestinal mucosa of mice immunized with DNA+Protein, DNA, and Vector alone compared to other groups after second immunization. Mice immunized with DNA+Protein (only one immunization of oral DNA) produced lower total sIgA compared to mice immunized with three immunizations of DNA or Vector alone, indicating that the total sIgA was induced mostly by attenuated *Salmonella* bacteria (**Figure 2A**). The anti-*Ts*Pmy specific sIgA was detected only in mice immunized with DNA and DNA+Protein. Mice immunized with once oral DNA followed by two boosts of protein (DNA+Protein) elicited the similar level of antigen-specific sIgA as mice immunized with oral DNA for three times (DNA) without significant difference. Mice immunized with protein (intramuscular) or empty vector did not induce any anti-*Ts*Pmy sIgA response (**Figure 2B**).

### Dynamic Cytokine Profiles

**FIGURE 3:**
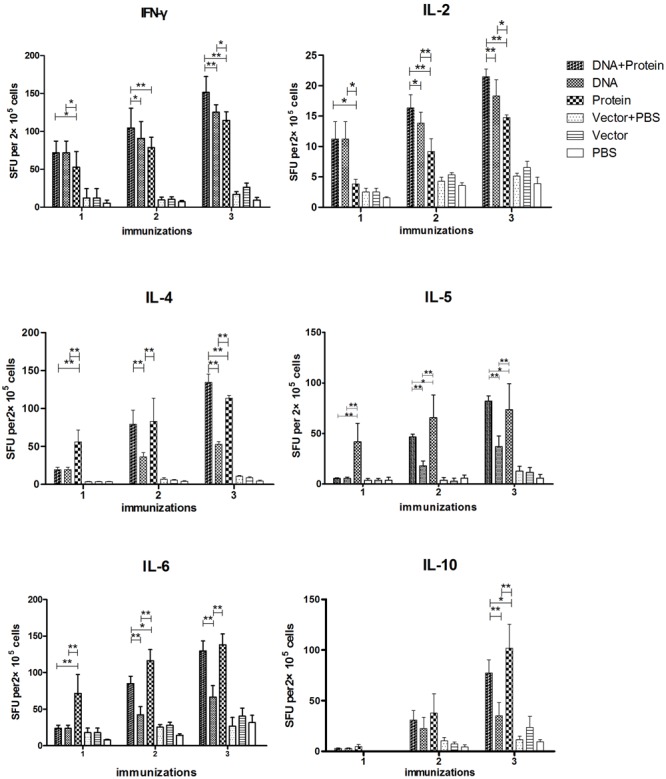
Dynamic cytokine profile secreted by splenocytes upon r*Ts*Pmy re-stimulation. Splenocytes secreting Th1 cytokines (IFN-γ and IL-2), Th2 cytokines (IL-4, IL-5, IL-6, and IL-10) upon re-stimulated with 1 μg/ml of r*Ts*Pmy for 48 h were detected by ELISPOT assays. The values shown for each group are the mean ± standard deviation (SD) of cytokine levels from five individual mice. ^∗^*p* < 0.05; ^∗∗^*p* < 0.01 between two groups.

**FIGURE 4:**
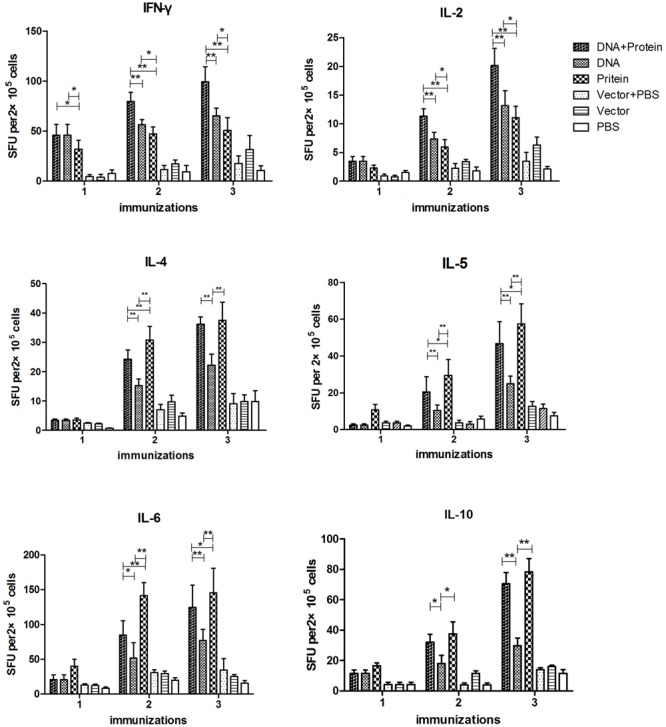
Dynamic cytokine profile secreted by mesenteric lymph nodes (MLNs) cells upon r*Ts*Pmy re-stimulation. MLN cells secreting Th1 cytokines (IFN-γ and IL-2), Th2 cytokines (IL-4, IL-5, IL-6, and IL-10) upon re-stimulated with 1 μg/ml of r*Ts*Pmy for 48 h were detected by ELISPOT assays. The values shown for each group are the mean ± standard deviation (SD) of cytokine levels from five individual mice. ^∗^*p* < 0.05; ^∗∗^*p* < 0.01 between two groups.

The cytokine responses including IFN-γ, IL-2, IL-4, IL-5, IL-6, and IL-10 in splenocytes (**Figure 3**) and MLN cells (**Figure 4**) were measured by ELISPOT 1 week after each immunization. All of the cytokines were significantly increased after the first immunization and reached the highest after three immunizations in mice immunized with DNA+Protein, DNA, or Protein alone in both splenocytes and MLN cells upon re-stimulation of r*Ts*Pmy. In the systematic splenocyte responses, mice immunized with DNA+Protein produced significant higher Th1 cytokines including IFN-γ and IL-2 than mice immunized with DNA or Protein alone, similar levels of Th2 cytokines (IL-4, IL-5, IL-6, and IL-10) to the mice immunized with recombinant protein (Protein). Even though mice immunized with DNA prime-boost produced significant level of Th1 cytokines (IFN-γ and IL-2), the level of Th2 cytokines (IL-4, IL-5, IL-6, and IL-10) were much lower than groups immunized with DNA+Protein or Protein alone (**Figure 3**). The similar pattern of cytokine profile was observed in the local MLN cell responses to the re-stimulation of r*Ts*Pmy, with high Th1 cytokine responses (IFN-γ and IL-2) in mice immunized with DNA prime-boost, high Th2 cytokine (IL-4, IL-5, IL-6, and IL-10) responses in mice immunized with Protein prime-boost, and with highest Th1/Th2 cytokine responses for mice immunized with DNA+Protein prime-boost (**Figure 4**). There was no significant cytokine response in both splenocytes and MLN cells from empty control mice. These results suggest that mice immunized with *Ts*Pmy using heterologous prime-boost immunization regimen induced highest Th1 and Th2 cytokine responses in both systemic lymphocytes (spleen) and local lymphocytes around the intestine (MLNs).

### Protective Immunity

**FIGURE 5:**
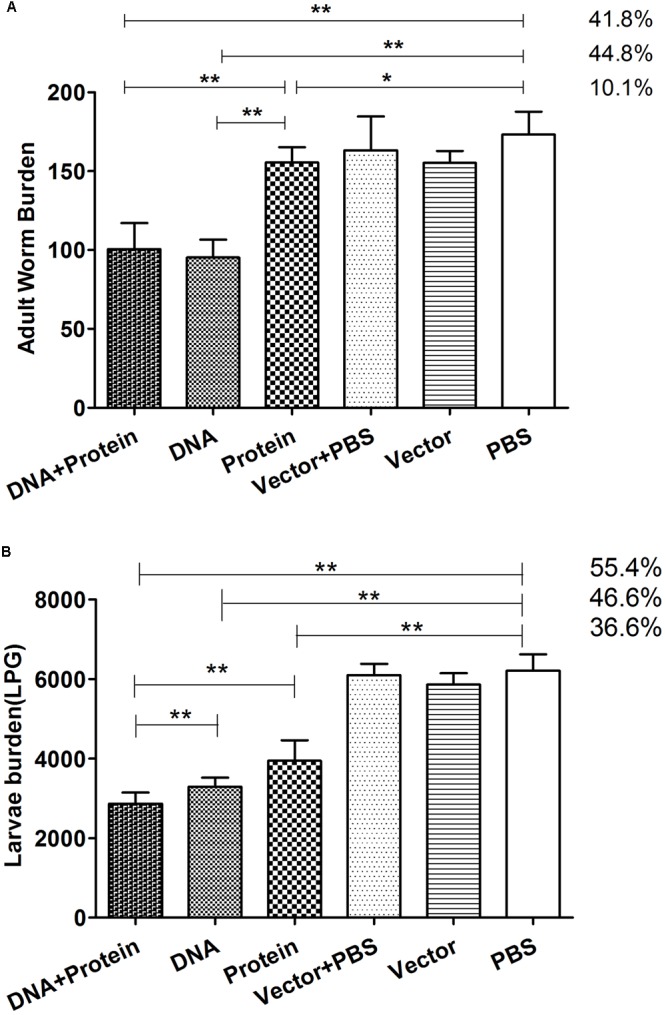
The adult worm **(A)** and larvae per gram (LPG) of muscle **(B)** collected from vaccinated mice after a challenge with 500 *Trichinella spiralis* infective larvae. The results are presented as the arithmetic mean ± standard deviation (SD) of 10 mice each group. ^∗^*p* < 0.05; ^∗∗^*p* < 0.01 between two groups.

Two weeks after final immunization with *Ts*Pmy in different regimens, six groups of mice were challenged orally with 500 ML per mouse. The worm burden results reveal that mice immunized with DNA+Protein prime-boost, DNA alone and Protein alone prime-boost produced 41.8, 44.8, and 10.1%, respectively, adult worm reduction compared with mice received PBS control (**Figure 5A**). Mice immunized with DNA+Protein and DNA alone prime-boost induced significant higher adult worm reduction than mice immunized with Protein alone prime-boost. Muscle larvae collection 42 days after challenge demonstrated that mice immunized with DNA+Protein prime-boost, DNA and Protein alone prime-boost produced 55.4, 46.6, or 36.6% muscle larvae reduction, respectively, compared to mice received PBS control (*p* < 0.01) (**Figure 5B**). The number of adult worms and ML collected from each group of mice was shown in Supplementary Table 1. The statistical analysis shows that mice immunized with *Ts*Pmy in DNA/protein heterologous prime-boost regimen produced significant higher larvae reduction than mice in groups of DNA prime-boost (*p* < 0.05) and protein prime-boost (*p* < 0.05), mice immunized with *Ts*Pmy in DNA prime-boost regimen produced higher larvae reduction than mice in protein prime-boost (*p* < 0.05). These adult and muscle larvae reduction results indicate that *Ts*Pmy immunization in DNA/protein heterologous prime-boost regimen induced significantly higher protective immunity than the DNA or protein homologous prime-boost regimens against *T. spiralis* infection in BALB/c mice, especially at the larvae reduction level.

## Discussion

In general, DNA vaccine and recombinant protein vaccine stimulates different antigen-specific immune response using different mechanisms, the former induce strong Th1 cellular immune responses and also prime antigen-specific memory B cells, and the latter induce predominant Th2 antibody responses (Jackson et al., 2014; Kardani et al., 2016). This different immunological mechanisms lead to the heterogeneous DNA-prime and protein-boost immunization regimen in order to enhance both cellular and humoral immune responses. DNA vaccine is used to prime immune responses including antigen-specific memory B cells. A protein vaccine is followed as boosting immunization to directly stimulate memory B cells to differentiate into antibody-producing cells (Alekseeva et al., 2009; Ranasinghe and Ramshaw, 2009). This heterologous prime-boost regimen has been proven to be more effective than homologous prime-boost approach (Nascimento and Leite, 2012; Rafferty et al., 2014). This type of heterologous prime-boost may elicit unique cellular and humoral immune responses as well as mucosal immune response to achieve improved and suitable immune responses to defense different pathogens including viral, bacterial, and parasitic infections (Butts et al., 2016; Vemula et al., 2016).

In our previous studies, immunization with r*Ts*Pmy protein, immunogenic epitope peptides (Wei et al., 2011; Gu et al., 2013), or *Ts*Pmy DNA vaccine delivered by attenuated bacteria vector (Wang et al., 2016) induced certain extent of protective immune responses against *Trichinella* infection. The previous vaccine efficacy comparison among available *Ts*Pmy-based *T. spiralis* vaccines was shown in Supplementary Table 2. However, the protection efficacy induced by these homologous prime-boost immunizations is insufficient (33.44–46.6% protection for ML). In this study, we report that a vaccination with heterologous prime-boost strategy comprised of oral prime inoculation with *Ts*Pmy DNA vaccine delivered by attenuated *S. typhimurium* (SL7207) and two intramuscular boosts with r*Ts*Pmy protein, strongly elicited both cell medicated immune responses and humoral immune responses. The robust balanced Th1/Th2 immune responses upon the *Ts*Pmy heterologous prime-boost immunization produced the optimal protective efficacy with 41.8% adult worm reduction and 55.4% larvae reduction in a mouse model compared to homologous regimens vaccinated with *Ts*Pmy DNA (44.8%/46.6% reduction for adult worm/ML) or Protein (10.1% and 36.6%) alone. In particular, *Ts*Pmy heterologous prime-boost immunization induced significant better reduction of muscle larvae, which cause major disease manifestation of trichinellosis in humans (Gottstein et al., 2009).

Mucosal immunity acts as the first barrier of defense against intestinal pathogens such as *T. spiralis*. It has been demonstrated that mucosal IgA response was able to impede the establishment of *Trichinella* adult worms in the mouse intestine (Ding et al., 2016). Previous study has demonstrated that oral immunization with *Ts*Pmy/*S. typhimurium* DNA vaccine induced *Ts*Pmy-specific sIgA secretion in intestinal mucosa possibly through inducing the expression of CCR9/CCR10, the homing receptors, on antibody secreting B cells and B cell translocation to local intestinal mucosa (Wang et al., 2016). In this study, one oral immunization with *S. typhimurium*-delivered *Ts*Pmy DNA in the heterologous DNA/prime and protein/boost regimen induced the similar level of antigen-specific sIgA (**Figure 2B**) in gut mucosa as the three immunizations of homologous DNA prime-boost regimen, indicating one oral immunization of *Ts*Pmy is potent to induce mucosal sIgA and the following boosted recombinant protein may stimulate the specific B memory cells in mucosa to produce sIgA, which also reflects the similar adult worm reduction in the gut between the groups of *Ts*Pmy heterologous DNA–protein and homologous DNA immunizations.

Indeed, oral immunization with *S. typhimurium*-delivered *Ts*Pmy DNA induced strong mucosa sIgA that may contribute to the protection against adult worm parasitism in the gut; however, mucosal sIgA alone is insufficient to provide solid protection against intestinal pathogen infections (Azegami et al., 2014; Zaph et al., 2014), especially has no effect on the newborn larvae that have already penetrated through mucosa and migrated to muscle to form muscle larvae. A systemic immune response, including humoral antibody and cellular response, is needed to defend invaded larvae (Yang et al., 2016). It is well-known that protein antigens are able to strongly boost systemic immune responses and a combination of DNA prime and protein-boost significantly augmented and elongated humoral and cellular responses to HIV Env (Jalah et al., 2014). In this study, significantly greater systemic humoral and cellular immune responses were observed in the heterologous DNA-prime and protein-boost immunization with *Trichinella* leading vaccine antigen *Ts*Pmy. In addition to the strong intestinal mucosal sIgA response, the anti-*Ts*Pmy specific IgG antibody was significantly increased after being boosted with r*Ts*Pmy protein and reached to peak value after the second boost with r*Ts*Pmy, which was even greater than protein prime-boost vaccination group. Cytokine profile of systemic lymphocytes (spleen) and intestinal local lymphocytes (MLN) upon re-stimulation of r*Ts*Pmy also showed that Th1 cytokines (IFN-γ, IL-2) was induced in DNA immunization, and significantly boosted by following intramuscular immunizations of r*Ts*Pmy to the level which is even higher than that boosted by DNA prime-boost vaccination. The heterologous DNA-prime and protein-boost immunization also boosted strong Th2 cytokines (IL-4, IL-5, IL-6, and IL-10) to the similar level as group in protein prime-boost regimen. Both humoral and cellular immune responses play important roles against pathogenic *T. spiralis* infections (Lee and Ko, 2006). The heterologous prime-boost regimen may contribute to the better protection against *Trichinella* infection than DNA or protein homologous prime-boost regimens, especially with significant better protection against the formation of muscle larvae.

## Conclusion

We demonstrate here that a heterologous immunization regimen with *Ts*Pmy DNA-prime orally delivered by attenuated *S. typhimurium* and protein-boost effectively induced mucosal sIgA response and an enhanced and balanced Th1/Th2 systemic immune responses that resulted in improved protection against *T. spiralis* infection, with 55.4% muscle larvae reduction which is significant higher than that induced by the homologous DNA or protein prime-boost regimen. Such an immunization strategy, plus combination with other protective antigen, would be a better approach to enhance protective immunity and could be used to better control trichinellosis in human and domestic animals.

## Author Contributions

XZ and LW conceived and designed the experiments. LW, XS, and JH performed the experiments. XZ, LW, and BZ analyzed the data. LW, XZ, and BZ wrote the paper. All authors reviewed the manuscript.

## Conflict of Interest Statement

The authors declare that the research was conducted in the absence of any commercial or financial relationships that could be construed as a potential conflict of interest.
