# Supplementary material for: Heterologous Prime-Boost Vaccination Enhances TsPmy’s Protective Immunity against Trichinella spiralis Infection in a Murine Model
Source: Front Microbiol. 2017 Jul 21;8:1394. doi: 10.3389/fmicb.2017.01394 (PMC5519575; doi:10.3389/fmicb.2017.01394)
Supplement: Supplementary file 1 [file Table_1.DOCX]

Supplementary Table 1. *T. spiralis* Adult worms and ML collected from each group of mice upon being challenged with 500ML each.

| Group | Adult worms# | Mean±SD | Reduction% | ML#（LPG） | Mean±SD | Reduction% |
| --- | --- | --- | --- | --- | --- | --- |
| DNA+Protein | 82,88,100,124,86,90,123,91,110,112 | 100.6±15.6 | 41.8%^**^ | 2844.1,2830.1,2639.2,2442.9,2862.4,2802.1,2855.4,2935,2862.3,2637.1 | 2771.1±150.4 | 55.4%^**^ |
| DNA | 88,111,98, 90,112,77, 87,105,98, 88 | 95.4±11.4 | 44.8 %^**^ | 3307.5,2569.6,3213.7,2810.6,2955.0,3870.9,3947.8,2693.8,3852.7,3940.8 | 3316.2±550.4 | 46.6%^**^ |
| Protein | 140,162,159,160,165,139,150,152,162,166 | 155.5±9.8 | 10.1%^**^ | 4002.7,3977.5,3824.6,4221.5,4238.1,3652.7,4228.9,3824.7,3747.5,3687.2 | 3940.5±227.8 | 36.6%^**^ |
| Vector+PBS | 188,148,144,169,184,171,174,156,150,148 | 163.2±16.0 | - | 5338.6,6447.9,5341.4,6445.1,6310.4,5985.6,6317.5,6321.1,5925.7,6524.5, | 6095.8±442.1 | - |
| Vector | 160,152,157,168,157,157,146,145,150,162 | 155.4±7.2 | - | 6620.2,5071.9,5354.4,6110.4,5350.7,6745.3,5263.4,6115.4,5198.7,6772.3 | 5860.2±687.3 | - |
| PBS | 163,162,181,160,174,181,176,150,188,195 | 173.0±14.0 | - | 5221.5,5709.7,5219.7,5929.7,5668.3,7653.1, 6947.5,5117.7,7334.7,7328.7 | 6213.0±996.3 | - |

^**^*p*<0.01 compared to PBS controls.
