# Supplementary material for: Heterologous Prime-Boost Vaccination Enhances TsPmy’s Protective Immunity against Trichinella spiralis Infection in a Murine Model
Source: Front Microbiol. 2017 Jul 21;8:1394. doi: 10.3389/fmicb.2017.01394 (PMC5519575; doi:10.3389/fmicb.2017.01394)
Supplement: Supplementary file 2 [file Table_2.DOCX]

Supplementary table 2. The vaccine efficacy comparison among available *Ts*Pmy-based *T. spiralis* vaccines developed in our lab.

| Vaccine name | Vaccine type | Adult worm reduction | ML reduction | Reference# |
| --- | --- | --- | --- | --- |
| *Ts*Pmy +ISA206 | Recombinant protein | - | 33.7% | (Yang et al., 2010) |
| *Ts*Pmy +ISA720 | Recombinant protein | - | 34.9% | (Yang et al., 2010) |
| *Ts*Pmy +Freund’s | Recombinant protein | - | 36.7% | (Yang et al., 2010) |
| *Ts*Pmy | Epitope (peptide) | - | 33.4% | (Wei et al., 2011) |
| *Ts*Pmy + *Ts*87 | Multi-epitopes (peptide) | - | 35.0% | (Gu et al., 2013) |
| *Ts*Pmy DNA | DNA/*Salmonella* | 44.8% | 46.6% | (Wang et al., 2016) |
| *Ts*Pmy Prime-Boost | DNA/*Salmonella+* Recombinant protein | 41.8% | 55.4% | Current study |

**References**

Gu, Y., Wei, J., Yang, J., Huang, J., Yang, X., and Zhu, X. (2013). Protective immunity against Trichinella spiralis infection induced by a multi-epitope vaccine in a murine model. *PLoS One* 8, e77238. doi: 10.1371/journal.pone.0077238

Wang, L., Wang, X., Bi, K., Sun, X., Yang, J., and Gu, Y. (2016). Oral Vaccination with Attenuated Salmonella typhimurium-Delivered TsPmy DNA Vaccine Elicits Protective Immunity against Trichinella spiralis in BALB/c Mice. *PLoS Negl Trop Dis* 10, e4952. doi: 10.1371/journal.pntd.0004952

Wei, J., Gu, Y., Yang, J., Yang, Y., Wang, S., and Cui, S. (2011). Identification and characterization of protective epitope of Trichinella spiralis paramyosin. *Vaccine* 29, 3162-3168. doi: 10.1016/j.vaccine.2011.02.072

Yang, J., Gu, Y., Yang, Y., Wei, J., Wang, S., and Cui, S. (2010). Trichinella spiralis: immune response and protective immunity elicited by recombinant paramyosin formulated with different adjuvants. *Exp. Parasitol.* 124, 403-408. doi: 10.1016/j.exppara.2009.12.010
